# Dynamic changes of hematological and hemostatic parameters in COVID-19 hospitalized patients: Potential role as severity biomarkers for the Chilean population

**DOI:** 10.5937/jomb0-47588

**Published:** 2024-06-15

**Authors:** Pablo Letelier, Hugo Delgado, Felipe Garrido, Francisco Quiñones, Martín Andrés San, Loreto Hernández, Paola Garcés, Dina Guzmán-Oyarzo, Rodrigo Boguen, Alfonso Hernandez, Gustavo Medina, Patricia Schwerter, Neftalí Guzmán

**Affiliations:** 1 Universidad Católica de Temuco, Facultad de Ciencias de la Salud, Departamento de Procesos Diagnósticos y Evaluación, Precision Health Research Laboratory, Temuco, Chile; 2 Dr. Hernán Henríquez Aravena Hospital, Clinical Laboratory, Temuco, Chile; 3 Complejo Asistencial Padre Las Casas, Padre Las Casas, Araucanía, Chile; 4 Red Salud Mayor, Temuco, Chile; 5 Universidad San Sebastián, Facultad de Medicina y Ciencias, School of Medical Technology, Campus Concepción, Concepción, Chile; 6 Universidad Católica de Temuco, Facultad de Ingeniería, Department of Mathematical and Physics Sciences, Temuco, Chile

**Keywords:** COVID-19, SARS-CoV-2, hematology, laboratory markers, prognosis, COVID-19, SARS-CoV-2, hematologija, laboratorijski markeri, prognoza

## Abstract

**Background:**

COVID-19 is still a global health issue, there is limited evidence in South America regarding laboratory biomarkers associated with severe disease. The objective of our study was to identify hematological and hemostatic changes associated with severe COVID-19.

**Methods:**

A total of 170 hospitalized patients with COVID19 were included in the study, defining their severity according to established criteria. Demographic, clinical, and laboratory (days 1, 3, 7, 15) data were obtained. We performed a statistical analysis, assuming significance with a value of p < 0.05. We analyzed the correlation between severity and biomarkers and established cut-off values for severe patients through ROC curves, estimating Odds Ratio associated with severe disease.

**Results:**

Day 1 was observed significant differences between moderate vs severe patients for leukocytes (WBC), Neutrophil-lymphocyte ratio (NLR), platelet-lymphocyte ratio (PLR) and D-dimer, establishing cut-off points for each of them. The markers we found associated to risk of severe disease were WBC (OR=3.2396; p = 0.0003), NLR (OR=5.7084; p < 0.0001), PLR (OR=4.4094; p < 0.0001), Neutrophil (OR=4.1193; p < 0.0001), D-dimer (OR=2.7827; p = 0.0124).

**Conclusions:**

The results allow to establish basic laboratory biomarkers associated to severe disease, which could be used as prognostic markers.

## Abstract (sr)

**Uvod:**

COVID-19 je i dalje globalno zdravstveno pitanje, u Južnoj Americi postoje ograničeni dokazi u vezi sa laboratorijskim biomarkerima povezanim sa teškom bolešću. Cilj naše studije je bio da se identifikuju hematološke i hemostatske promene povezane sa teškim COVID-19.

**Metode:**

U studiju je uključeno ukupno 170 hospitalizovanih pacijenata sa COVID-19, definišući njihovu težinu prema utvrđenim kriterijumima. Dobijeni su demografski, klinički i laboratorijski (1., 3., 7., 15. dan) podaci. Izvršili smo statističku analizu uz pretpostavku da je značajna vrednost p < 0,05. Analizirali smo korelaciju između težine i biomarkera i utvrdili granične vrednosti za teške pacijente kroz ROC krive, procenjujući Odds Ratio povezan sa teškom bolešću.

**Rezultati:**

Prvi dan primećene su značajne razlike između umerenih i teških pacijenata za leukocite (VBC), odnos neutrofila-limfocita (NLR), odnos trombocita-limfocita (PLR) i Ddimer, uspostavljajući granične tačke za svaki od njih. Markeri za koje smo otkrili da su povezani sa rizikom od teške bolesti bili su VBC (OR=3,2396; p = 0,0003), NLR (OR= 5,7084; p < 0,0001), PLR (OR=4,4094; p < 0,0001), Neutrofil (OR=4,1193; p < 0,0001), D-dimer (OR=2,7827; p = 0,0124).

**Zaključak:**

Rezultati omogućavaju uspostavljanje osnovnih laboratorijskih biomarkera povezanih sa teškim oboljenjem, koji se mogu koristiti kao prognostički markeri.

## Introduction

Coronavirus disease 2019 (COVID-19), declared a pandemic in March 2020, remains a global public health problem, causing more than 6.6 million deaths worldwide.

As a result of the rapid spread and burden of disease of the novel coronavirus, researchers and pharmaceutical companies had to develop vaccines using preexisting or novel technologies rapidly [1]. Although progress has been made in the vaccination strategy, outbreaks continue to persist worldwide that are associated with severe disease and mortality.

The clinical features of coronavirus infection vary widely, from asymptomatic infection to severe pneumonia with respiratory failure and even death [2]. Fever and coughing are the most common symptoms at a global level [3]. Moreover, there are still many unknowns in the current understanding of the pathogenesis of prolonged COVID, some factors associated with the pathophysiology are vaccination, viral variants, the environment and social factors associated with the host [4]. In combination with epidemiological history and clinical symptoms, together with [5] [6] reliable techniques for COVID-19 diagnosis as radiological examinations and molecular and immunological techniques (genome sequencing, Nucleic Acid Amplification Tests, clustered regularly interspaced short palindromic repeats (CRISPR), antigen/antibody detection), it has been possible to identify and prevent new COVID-19 infections [7] [8].

In addition, the routinely studied biomarkers in Clinical laboratories can play a fundamental role in improving the diagnosis, and prognosis, allowing patients to be categorized into different medical units, in addition to monitoring the evolution towards more severe forms of the disease, supporting the various therapeutic strategies [9] [10] [11] [12] [13]. Laboratory findings may be normal, as well as decreased leukocyte count, decreased lymphocyte count, thrombocytopenia, increased transaminases, increased lactate dehydrogenase (LDH), creatine kinase-myoglobin elevation [14] C Reactive Protein (CRP) and Total bilirubin and the decrease in albumin [11]. Accumulated evidence demonstrates that haematological parameters can be altered in COVID-19 patients, being able to become potential biomarkers of prognosis and monitoring of treatment, presenting dynamic changes in the course of the disease [15] [16] [17] [18] [19]. Nevertheless, available evidence in South America is limited. Thus, the objective of this study was to identify haematological and hemostatic changes associated with illness severity in COVID-19 patients.

## Materials and methods

### Study design and participants

By way of a retrospective study, a total of 170 COVID-19 diagnosed hospitalized patients (> 18 years old), at the Dr. Hernán Henríquez Aravena Hospital in Temuco, Chile, were included in this study. The patients were diagnosed in accordance with the established criteria, being confirmed by reverse transcriptase Real Time Polymerase Chain Reaction (qRT-PCR) of nasal and pharyngeal swab specimens. The study was approved by the Scientific Ethical Committee (N° 144/2020) and was performed in accordance with the Helsinki Declaration ethical norms.

### Data collection

COVID-19 severity was defined as a moderate to severe disease according to the WHO *Clinical Progression Scale* [20]. Epidemiological, demographic, and clinical data were obtained from each patient s medical history. Demographic variables included age and sex, while the medical history considered comorbidities such as diabetes, arterial hypertension, obesity, heart disease, chronic respiratory pathologies, chronic kidney disease, and chronic hepatic disease.

On the other hand, we registered haematology and haemostatic laboratory results obtained from the laboratory information system (LIS), at days 1, 3, 7 and 15 of hospitalization. All the samples for day 1 were collected within the first 24 h of hospital admission. The results were obtained by complete and differential blood count of leukocyte population through haematology analyser MINDRAY CAL 6000 (Mindray, China), obtaining the neutrophil-lymphocyte ratio (NLR) and platelet-lymphocyte ratio (PLR). Haemostasis tests (prothrombin time, activated partial thromboplastin time, and D-dimer were performed by clotting time (PT and APTT) and immunoturbidimetric (D-dimer) assays, using a STA®R Max coagulation analyser (Stago, Asnières sur Seine, France).

### Statistical analysis

We performed a statistic analysis using SPSS 24.0 (SPSS Inc., Chicago, IL, USA), considering as statistically significant a value of p <0.05. For qualitative variables we performed a nonparametric Pearson Chi-Square test [2]. Continuous variables were expressed as means and standard deviations, for the comparison between two groups the parametric t-Student test was applied in the case of normal distributions or the non-parametric Mann-Whitney test for non-normal distributions. For correlation analysis, Spearman's correlation coefficient was used, while to determine the optimal cut-off points in severe patients, analysis was performed using ROC (Receiver–operating characteristic) curves. Finally, *Odds Ratio* was calculated to establish severity risk.

## Results

From the total of patients included in this study, 104 presented moderate disease, while 66 presented severe disease. The demographic and clinical characteristics of patients are presented in Table 1. Of the patients who presented previous health conditions, we observe significant differences pertaining obesity (*p* < 0.001) and chronic hepatic disease (*p*= 0.01). On the other hand, the most frequent clinical manifestations corresponded to cough, fever, dyspnea and myalgia, observing significant differences for dyspnea (p=0.006) and gastrointestinal symptoms (*p*=0.042).

**Table 1:** Demographic and clinical characteristic of Chilean patients hospitalized with COVID-19. P values indicate differences between moderate and severe. SD= Standard Deviation

Characteristic	Total<br>(n= 170)<br>No (%)	Moderate<br>(n= 104)<br>No (%)	Severe<br>(n= 66)<br>No (%)	p Value
*Demographics*				
Sex				
Male	82 (48.2)	50 (48.1)	32 (48.5)	0.99
Female	88 (51.8)	54 (51.9)	34 (51.5)	
Age (years)				
Average ± SD	59.7±14.5	59.2±14.9	60.4±13.9	0.51
*Comorbidities*				
Arterial Hypertension	93 (54.7)	51 (49)	42 (63.4)	0.08
Obesity	60 (35.3)	26 (25)	34 (51.5)	< 0.001
Diabetes Mellitus 2 (DM2)	58 (34.1)	30 (28.8)	28 (42.4)	0.09
Cardiovascular Disease	26 (15.3)	17 (16.3)	9 (13,6)	0.8
Chronic Respiratory Pathology	21 (12.4)	10 (9.6)	11 (16.7)	0.23
Chronic Obstructive Pulmonary (COPD)	8 (4.7)	2 (1.92)	6 (9.1)	0.06
Chronic Renal Disease	15 (8.8)	6 (5.8)	9 (13.6)	0.09
Chronic Hepatic Disease	7 (4.1)	1 (.96)	6 (9.1)	0.01
*Clinical Manifestations*				
Fever	101 (59.4)	57 (54.8)	44 (66.7)	0.06
Odynophagia	31 (18.2)	19 (18.3)	12 (18.2)	0.91
Gastrointestinal Symptoms	38 (22.4)	29 (27.9)	9 (13.6)	0.04
Cough	111 (65.2)	65 (62.5)	46 (69.7)	0.15
Headache	44 (25.9)	31 (29.8)	13 (19.7)	0.19
Dyspnea	116 (68.2)	64 (61.5)	52 (78.8)	0.006
Myalgia	61 (35.9)	36 (34.6)	25 (37.9)	0.52
Fatigue	30 (17.6)	19 (18.2)	11 (16.7)	0.89
Anosmia	11 (6.5)	6 (5.8)	5 (7.6)	0.59
Dysgeusia	11 (6.5)	8 (7.7)	3 (4.5)	0.46

### Haematological and haemostatic changes

To determine potential severity-related biomarkers we analysed laboratory parameters at days 1, 3, 7 and 15 of hospitalization, which can be viewed on Table 2. When comparing the results of haematological parameters between moderate and severe patients at admission (day 1), we observed significant differences for WBC (7.4 ± 3.16 x 10^9^/L moderate vs 9.1 ± 3.37 x 10^9^/L severe, p < 0.01), NLR (4.9 ± 3.55 moderate vs 10.7 ± 7.66 severe, p < 0.01), PLR (202.7 ± 124.02 moderate vs 328.1 ± 235.31 severe, p < 0.01). For leukocyte populations, significant differences were observed in lymphocytes (1.33 ± 0.60 x 10^9^/L moderate vs 0.98 ± 0.67 x 10^9^/L severe, p < 0.01), neutrophils (5.42 ± 2.94 x 10^9^/L moderate vs 7.46 ± 3.07 x 10^9^/L severe, p < 0.01), eosinophils (0.06 ± 0.15 x 10^9^/L moderate vs 0.02 ± 0.06 x 10^9^/L severe, p < 0.01) and basophils (0.01 ± 0.03 x 109/L moderate vs 0.0027 ± 0.011 x 109/L severe, p < 0.05). Additionally, significant differences were observed for D-dimer (1.22 ± 0.99 μg/mL moderate vs 2.07 ± 2.10 μg/mL severe, p < 0.05). Positive correlation was found between disease severity and WBC, neutrophils, NLR, PLR and D-dimer, while a negative correlation was observed with lymphocytes, eosinophils, and basophils (Table 3).

**Table 2:** Hematological and hemostatic parameters in patients hospitalized with COVID-19. Abbreviations: RBC=Red blood cell; WBC=White blood cell; PLT=Platelets; MCV=Mean corpuscular volume; MCH=Mean corpuscular hemoglobin; MCHC=Mean corpuscular hemoglobin concentration; RDW=Red Cell Distribution Width; NLR=Neutrophil-Lymphocyte Ratio; PLR=platelet-lymphocyte ratio; APTT= Activated partial thromboplastin time; PT=Prothrombin time. P values indicate differences between moderate and severe. *p< 0,05; **p< 0,001.

PARAMETER	REFERENCE<br>INTERVAL	DAY 1		DAY 3		DAY 7		DAY 15	
		MODERATE<br>(X ± SD)	SEVERE<br>(X ± SD)	MODERATE<br>(X ± SD)	SEVERE<br>(X ± SD)	MODERATE<br>(X ± SD)	SEVERE<br>(X ± SD)	MODERATE<br>(X ± SD)	SEVERE<br>(X ± SD)
Hematocrit,<br>L/L	35.0–47.0	37.8±5.8	36.7±6.40	36.8±6.23	35.1±5.29	36.6±6.87	33.4±5.27**	32.7±5.97	32.0±4.59
Hemoglobin,<br>g/L	140–175	127±22.5	123±23.8	124±22.4	116±18.2*	123±24.3	109±19.0**	108±20.6	104±16.1
RBC, 10^12^/L	3.8–5.8	4.3±0.06	4.2±0.074	4.2±0.76	3.9±0.62*	4.1±0.83	3.8±0.99*	3.6±0.69	3.5±0.55
WBC, 10^9^/L	4.0–12.0	7.4±3.16	9.1±3.37**	7.4±3.29	9.8±4.67**	7.9±2.99	10.6±4.85**	8.1±3.14	10.1±3.18*
PLT, 10^9^/L	150–450	231.8±113.33	230.1±92.72	292.7±138.96	246.3±93.07*	333.2±137.19	282.3±112.02*	295.3±136.55	274.5±138.12
MCV, fL	82.0–95.0	87.9±5.69	88.4±6.11	88.1±4.96	89.8±5.48*	88.7±4.90	90.5±5.54	90.5±5.48	91.2±5.09
MCH, pg	25.0–32.0	29.6±2.35	29.7±2.38	29.5±1.99	29.7±2.02	29.7±1.96	29.3±3.17	29.9±2.36	29.7±2.01
MCHC, g/L	320–360	337±15.1	335±14.2	336±10.3	331±11.8**	334±10.9	327±12.9**	331±8.10	326±11.7
RDW, %	11.0–16.0	13.8±1.46	13.8±1.63	13.8±1.57	13.9±1.45	14.2±4.05	14.7±4.11	16.5±7.05	14.7±2.29
NLR	0.107–3.19	4.9±3.55	10.7±7.66**	5.2±4.34	13.4±1.68**	4.7±4.23	12.6±8.74**	5.6±5.42	9.4±6.83*
PLR	46.79–218.01	202.7±124.02	328.1±235.31**	150.6±16.53	291.9±36.49**	255.2±152.53	399.5±276.26**	276.6±240.94	288.9±167.44
Lymphocytes,<br>10^9^/L	0.84–4.2	1.33±0.60	0.98±0.67**	1.39±1.09	0.85±0.66**	1.53±0.62	0.89±0.43**	1.34±0.57	1.15±0.58
Monocytes,<br>10^9^/L	0.16–0.96	0.52±0.28	0.65±1.07	0.52±0.27	0.55±0.29	0.61±0.28	0.70±0.42	0.61±0.28	0.69±0.32
Neutrophils,<br>10^9^/L	2–8.2	5.42±2.94	7.46±3.07**	5.41±3.09	8.24±4.76**	5.69±2.99	8.84±4.52**	5.89±2.92	7.99±2.87*
Eosinophils,<br>10^9^/L	0.08–0.6	0.06±0.15	0.02±0.06**	0.09±0.16	0.05±0.11	0.10±0.13	0.07±0.13	0.19±0.21	0.93±5.78
Basophils,<br>10^9^/L	0–0.12	0.01±0.03	0.0027±0.011*	0.09±0.71	0.006±0.01	0.01±0.021	0.015±0.07	0.010±0.022	0.019±0.035
Prothrombin<brr>Time, %	70–100	89.0±17.98	85.4±18.76	85.0±15.95	84.5±18.005	87.5±16.34	81.4±12.33*	85.6±12.38	79.0 ±12.33
APTT, sec	26.3–40.3	32.6±6.10	34.5±7.83	32.0±6.05	36.6±18.96	31.1±6.55	32.4±5.39	29.9±4.44	33.8±7.63
D-dimer,<br>μg/mL	≤ 0.50	1.22±0.99	2.07±2.10*	1.30±1.27	2.67±3.37**	1.67±2.68	8.18±32.73	1.39±0.99	2.27±-1.57

**Table 3:** Correlation coefficient and P value between laboratory parameters and COVID-19 severity.

Laboratory<br>Parameters	r	P
WBC	0.2794	0.0002
Lymphocytes	-0.3468	< 0.0001
Neutrophils	0.3639	< 0.0001
Eosinophils	-0.2318	0.0024
Basophils	-0.1819	0.0176
NLR	0.4755	< 0.0001
PLR	0.2048	0.0074
D-dimer	0.2464	0.0085

**Figure 1:**
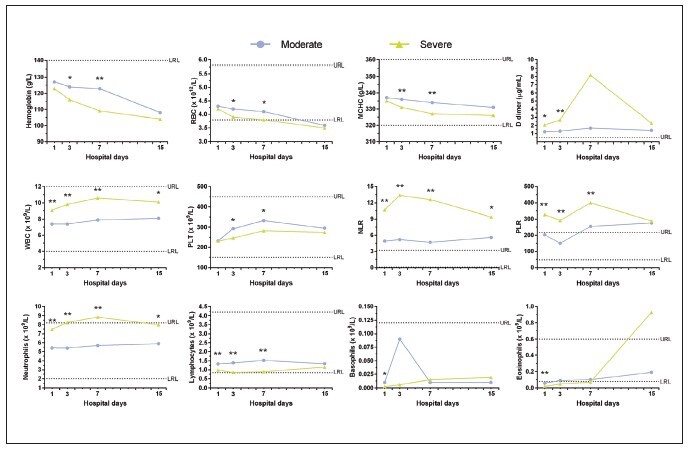
Dynamic variations of hematologic and hemostatic parameters in COVID-19 hospitalized patients. Abbreviations: RBC=Red blood cell; WBC=White blood cell; PLT=Platelets; MCHC= Mean corpuscular haemoglobin concentration; NLR= Neutrophil-Lymphocyte Ratio; PLR= platelet-lymphocyte ratio; LRL=Lower reference limit; URL= Upper reference limit. *p< 0,05; **p< 0,001.

From the third day of hospitalization, we observed significant differences for platelets (292.7 ± 138.96 x 10^9^/L moderate vs 246.3 ± 93.07 x 10^9^/L severe, p<0.05), and parameters such as haemoglobin (124 ± 22.4 g/L moderate vs 116 ± 18.2 g/L severe, p < 0.05) , RBC (4.2 ± 0.76 x 10^12^/L moderate vs 3.9 ± 0.62 x 10^12^/L severe, p < 0.05), MCV (88.1 ± 4.96 fL moderate vs 89.8 ± 5.48 fL severe, p < 0.05), MCHC (336 ± 10.3 g/L moderate vs 331 ± 11.8 g/L severe, p < 0.01), which show this trend until day 7 of hospitalization. Figure 1 presents the dynamic variations of laboratory parameters that showed significant differences between moderate vs. severe patients.

**Figure 2:**
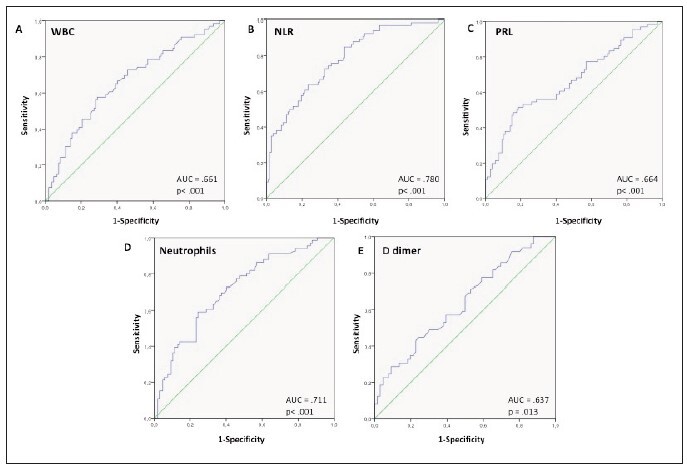
ROC curves of hematologic and haemostatic biomarkers in severe COVID-19 patients. A. WBC=White blood cell; B. NLR= Neutrophil-Lymphocyte Ratio; C. PLR= platelet-lymphocyte ratio; D. Neutrophils; E. D-dimer

The ROC curve analysis was used to determine optimal cut-off points in severely ill patients on hospital admission (Figure 2). The ROC curve for WBC (Figure 1A) shows that the optimal cut-off point was 7.905 x 10^9^/L (AUC= 0.661, CI 0.557–0.745, p<0.001) with a sensitivity of 57.6% and a specificity of 71%. For NLR (Figure 1B), the optimal cut-off point was 4.359 (AUC=0.780 CI 0.711–0.850, p<0.001) with a sensitivity of 84.8% and a specificity of 56.1%. For PLR (Figure 1C) the optimal cut-off point was 274.21 (AUC= 0.664, CI 0.579–0.750, p < 0.001) with a sensitivity of 51.5% and a specificity of 81.3%. The ROC curve for Neutrophils (Figure 1D) shows that the cut-off point was 6.26 x 10^9^/L (AUC= 0.711, CI 0.633–0.790, p < 0.001) with a sensitivity of 59.1% and a specificity of 75.7%. Finally, for D-dimer (Figure 1E) the optimal cut-off point was 1.38 mg/mL (AUC= 0.637, CI 0.535–0.738, p < 0.013) with 44.9% sensitivity and 75.8% specificity. The markers that were associated with the risk of severe disease corresponded to WBC (OR=3.2396; CI 1.7022–6.1656; p, 0.0003), NLR (OR=5.7084; CI 2.7084–12.3991; p<0.0001), PLR (OR=4.4094; CI 2.2152–8.7611; p<0.0001), Neutrophils (OR=4.1193; CI 2.1334 – 7.9541; p<0.0001), D-dimer (OR=2.7827; CI 1.2474–6.2078; p =0.0124). In patients who present the 5 altered parameters, a significant risk of progressing to severe conditions was observed (OR= 27.9278; CI 1.5971 – 488.3650; p < 0.0226).

## Discusion

COVID-19 is a global health problem, with an important number of confirmed cases and more than 6.6 million deaths around the world [1]. In Chile and South America there is limited evidence regarding laboratory biomarkers useful to establish prognostics of severe sickness. In this study, we analysed haematological and haemostatic parameters associated with severe COVID-19 in a group of 170 patients (104 moderated and 66 severe), who were treated in a public hospital in the south of Chile. In the group of patients included in this study, we did not observe significant differences related to age or gender, in contrast with other studies, where older age, sex are important predictors of disease severity [21] [22] [23]. The most frequent comorbidities were hypertension, obesity, and diabetes, similarly to what has been described for other populations [24] [25] [26] [27] [28] [29]. Nevertheless, of the studied comorbidities, we observed significant differences between moderated vs severe patients for obesity and chronic hepatic disease. In regards to obesity, results are consistent with available evidence that demonstrate its link with severity and mortality in COVID-19 [30] [31] [32]. A recent meta-analysis in 3,140,413 patients, show that obesity was associated with an increased risk of severe disease (RR=1.52, 95% CI 1.41–1.63, p<0.001) and high mortality (RR= 1.09, 95% CI 1.02–1.16, p=0.006) [33]. In the same way, it has been demonstrated a strong association between body mass index (BMI) and respiratory failure or admittance to critical patient unit [33] [34], observing that 90% of COVID-19 patients with respiratory insufficiency had a BMI higher than 25 kg/m^2^ and a mean BMI of 30 kg/m^2^ [35].

Diverse laboratory parameters have been associated with risk of unfavorable progression of the disease [36]. The hemogram results show that at the day of admission, severe patients present significant variations in WBC, NLR and PLR. For WBC, the results are consistent with evidence that shows changes in leukocyte count [24] [37] [38], changes that are maintained until day 15 of hospitalization. A study performed to Chilean population at the beginning of the pandemic showed alterations in haematological parameter such as lymphocyte counts, neutrophils, NLR and D-dimer in patients that are admitted to critical patient units, however, it did not demonstrate association with WBC and PLR [39]. Regarding NLR and PLR, it has been proposed the usefulness of these systemic inflammatory indicators as early markers of severity risk [19] [40] [41] [42]. The results show that for NLR the differences remain constant until day 15 of hospitalization, while for PLR the differences are maintained until day 7 of hospitalization. A recent study shows that NLR is associated with higher severity risk (OR 2.886, IC 2.064–4.860, *p*=0.019) [43] and NLR has important diagnostic value for differentiating COVID-19 patients from healthy subjects [42]. On the other hand, when analysing changes in PLR during treatment, Qu *et al.* propose that changes in PLR show the disease progression and prognostic of patients with COVID-19 [44].

The results show dynamic changes in platelets and red series parameters (Haemoglobin, RBC and MCHC) from day 3 of hospitalization, and that they are maintained until the seventh day, what could be related to the progression of the disease. For platelets, severe patients show lower platelet count compared to subjects with moderate disease. A meta-analysis of 1779 patients showed that a lower platelet count is associated to an increase of risk of severe disease and mortality in patients [45]. These findings could be explained, at least in part, by three mechanisms that explain platelet decrease in COVID-19 patients: Inhibition of thrombopoiesis by direct cytotoxic action of the virus on hematopoietic cells, destruction of platelets by the immune system, consumption of platelets by formation of microthrombus in the lung [46]. For the red series, the presence of anaemia or changes in RBC and haemoglobin are not frequent findings.

In a multicenter study in 1099 patients, Guan et al. [47] demonstrated that the haemoglobin levels are lower in severe vs non-severe patients. Decrease in haemoglobin can be a consequence of inflammatory changes because of the infection of SARS-CoV-2 which could interfere with erythropoiesis. Some authors propose that the low incidence of anaemia would be related to the presence of compensatory mechanisms induced by hypoxia, so that the decrease in haemoglobin levels could be an indicator of disease progression [48] [49]. Interestingly, although no differences in haemoglobin were observed between moderate vs. severe patients at day 15, there was a decrease in this haematological parameter in both groups.

Regarding blood hemostasis parameters, the most relevant finding is the D-dimer elevation in all hospitalized patients, observing that severe patients present significantly higher concentrations compared to individuals with moderate disease. These findings correlate with what is described in the literature regarding the role of D-dimer as a marker of severity and mortality in COVID-19 [50] [51] [52] [53]. A meta-analysis showed that D-dimer levels can distinguish severe COVID-19 patients with only moderate accuracy, as indicated by pooled sensitivity and specificity of 77% and 71% respectively, and AUC 77% [54]. Various coagulation abnormalities have been described associated with severe disease, with a wide variety of clinical presentations such as disseminated intravascular coagulation and thrombosis. Of the markers of hemostasis, D-dimer is widely used as a biomarker of coagulation and activation of fibrinolysis and some studies have shown that its peak would occur approximately on day 5 of hospitalization, being higher in patients in critical condition and unfavourable course of the disease [12]. The results allow establishing optimal cut-off points for laboratory parameters at hospital admission. Using these cut-off points, a significant association with severe disease was observed for CBC parameters such as WBC, NLR, PLR, absolute neutrophil count, and D-dimer. In patients with abnormalities in all of these laboratory parameters, a high risk was observed to progress to severe disease.

## Conclusion

The results of this study allow, for the first time in the Chilean population, to identify early haematological biomarkers associated with severe COVID-19. These markers, based on basic laboratory tests such as blood count and D-dimer, can be performed at the time of hospital admission, in any health centre, regardless of the level of complexity. Thus, the adequate stratification of patients would allow the health team to monitor the progression of the disease and improve clinical decision-making.

## Dodatak

### Acknowledgments

This work was supported by Vicerrectoría de Investigación y Postgrado, Universidad Católica de Temuco, [grant number 2023FIAS-NG-04 and VIPUCT 2023FIAS-PL-03].

### Conflict of interest statement

All the authors declare that they have no conflict of interest in this work.

### List of abbreviations

NLR, Neutrophil-lymphocyte ratio;<br>PLR, platelet-lymphocyte ratio;<br>COVID-19, Coronavirus disease 2019;<br>qRT-PCR, Real Time Polymerase Chain Reaction;<br>PT, The prothrombin time;<br>APTT, test activated partial thromboplastin time;<br>ROC Receiver-operating characteristic;<br>SD, Standard Deviation;<br>RBC, Red blood cell;<br>WBC, White blood cell;<br>PLT, Platelets;<br>MCV, Mean corpuscular volume;<br>MCH, Mean corpuscular hemoglobin;<br>MCHC, Mean corpuscular hemoglobin concentration;<br>RDW , Red Cell Distribution Width;<br>LRL, Lower reference limit;<br>URL, Upper reference limit;<br>LDH, Lactate dehydrogenase; <br>CK, creatine kinase;<br>CRP, C Reactive Protein;<br>BMI, body mass index
